# Enhanced Carrier Transport Performance of Monolayer Hafnium Disulphide by Strain Engineering

**DOI:** 10.3390/nano14171420

**Published:** 2024-08-30

**Authors:** Yun-Fang Chung, Shu-Tong Chang

**Affiliations:** Department of Electrical Engineering, National Chung Hsing University, Taichung 402202, Taiwan; lily840408@gmail.com

**Keywords:** mobility, strain, Kubo–Greenwood mobility approach, hafnium disulphide (HfS_2_)

## Abstract

For semiconducting two-dimensional transition metal dichalcogenides (TMDs), the carrier transport properties of the material are affected by strain engineering. In this study, we investigate the carrier mobility of monolayer hafnium disulphide (HfS_2_) under different biaxial strains by first-principles calculations combined with the Kubo–Greenwood mobility approach and the compact band model. The decrease/increase in the effective mass of the conduction band (CB) of monolayer HfS_2_ caused by biaxial tensile/compressive strain is the major reason for the enhancement/degradation of its electron mobility. The lower hole effective mass of the valence bands (VB) in monolayer HfS_2_ under biaxial compressive strain improves its hole transport performance compared to that under biaxial tensile strain. In summary, biaxial compressive strain causes a decrease in both the effective mass and phonon scattering rate of monolayer HfS_2_, resulting in an increase in its carrier mobility. Under the biaxial compressive strain reaches 4%, the electron mobility enhancement ratio of the CB of monolayer HfS_2_ is ~90%. For the VB of monolayer HfS_2_, the maximum hole mobility enhancement ratio appears to be ~13% at a biaxial compressive strain of 4%. Our results indicate that the carrier transport performance of monolayer HfS_2_ can be greatly improved by strain engineering.

## 1. Introduction

Since the thickness of TMDs can be reduced to less than a few nanometers, TMDs are a promising alternative channel of materials for achieving ultra-thin body field effect transistors (FETs) that are robust to short channel effects. TMDs can be classified into two types based on their crystal structures: trigonal prismatic (2H-TMD type) and octahedral (1T-TMD type). MoS_2_ is a typical TMD with a 2H-TMD type structure. TMDs with a 1T-TMD type (group space P3m1) structure have much lower lattice thermal conductivity and better thermoelectric performance at room temperature. This implies that HfS_2_ is a good 2D thermoelectric material. Hao Wang et al. predicted that the lattice thermal conductivity of bulk HfS_2_ is much lower than that of MoS_2_, making the HfS_2_ system a promising thermoelectric material [1]. They also investigated the thermoelectric properties of monolayer HfS_2_ and demonstrated that it is an excellent n-type thermoelectric material, with much improved thermoelectric performance when compared with the bulk. Furthermore, the valence band valleys of monolayer HfS_2_ can be tuned by external biaxial tensile strain owing to the degeneracy of the valence band valleys reaching a maximum at a strain value of 6%. Monolayer HfS_2_, like the CdI_2_-type structure, has become well-known due to its low lattice thermal conductivity resulting in promising applications in thermoelectric engineering [2]. Hongyan Guo et al. performed a systematic first-principles study of the effect of tensile strains on the electronic properties of early monolayer TMDs and suggested that tensile strain could significantly affect the electronic properties of many early TMDs in general, and the electronic bandgap in particular [3]. The bandgap of group IVB TMDs such as HfS_2_ increased as biaxial tensile strain and started to decrease at a strain of 6~8%. Their investigation suggested that strain engineering was an effective approach for modifying the electronic properties of TMDs such as monolayer HfS_2_, thereby opening an alternative way for future electronic applications. Recently, Mayuri Sritharan et al. pointed out that transition metal dichalcogenide (TMD) monolayers with native high-κ oxides presented a new avenue for the development of new generation field-effect transistor (FET) devices [4]. These TMD materials were experimentally shown to form a natively compatible high-dielectric oxide layer, which could enhance both transistor device scaling and supply voltage shrinking. They found that monolayer hafnium disulphide (HfS_2_) exhibited isotropic transport at a channel length of 10 nm with ON currents over 1000 μA/μm and good subthreshold swing and identified monolayer HfS_2_ as a superior material within the TMD family for ultra-scaled FETs.

Dr. Afzalian et al. from imec developed an atomistic-modelling solver (ATOMOS) to assess the physics and fundamental performance potential of a monolayer HfS_2_ FET down to a gate length of 5 nm, including the effect of carrier phonon scattering [5,6]. They predicted that monolayer HfS_2_ FET had good scalability down to a gate length of 5 nm, with a promising high on the current level. They also proposed the concept of a dynamically doped 2D FET, which scaled better than other 2D FET counterparts. Used in combination with a best mobility material such as monolayer HfS_2_, it allowed for the maintenance of excellent performance on the drive current and competitive energy-delay. In Reference [7], Dr. Jiwon Chang investigated the ballistic transport characteristics of monolayer HfS_2_ FET via quantum transport simulation. He focused on the role of degenerate conduction band (CB) valleys in monolayer HfS_2_ and predicted that the effect of channel orientation on device performance was much weaker in monolayer HfS_2_ FET owing to the degenerate CB valleys of monolayer HfS_2_.

Kanazawa et al. first experimentally evaluated the FET performance of HfS_2_ and revealed its potential for transistor channels [8]. Although the other properties of HfS_2_ were not thoroughly investigated, they also reported some basic characteristics of HfS_2_ essential for FET device fabrication. Continuous efforts to seek other 2-D materials led to the discovery of anisotropic 2-D materials. Unlike Mo-based TMDs, HfS_2_ is characterized by its highly anisotropic CB. Theoretical investigations of HfS_2_ FETs have been performed [5,6,7]. Anisotropic 2-D materials other than phosphorene, such as Hf-based-TMDs, have been explored with density functional theory (DFT) calculations [9,10]. Monolayers of Hf-based TMDs such as HfS_2_ are quite different from those of Mo-based TMDs because of different crystal symmetry and atomic bonding. They have indirect bandgaps with three-fold degenerate conduction band (CB) valleys whose dispersions around the minimum CB are highly anisotropic. Among Hf-based-TMDs, monolayer HfS_2_ exhibits sizable bandgaps, larger than 1 eV, suitable for nanoscale FET applications. In this study, we present a comprehensive computational study of the carrier mobility of monolayer HfS_2_ based on the first principle method, compact band model, and Kubo–Greenwood mobility approach. We discuss the influence of the band structure anisotropy of monolayer HfS_2_ on its carrier transport performance, as well as the strain engineering of monolayer HfS_2_.

As a member of 1T-MX_2_, the carrier transport performance of monolayer HfS_2_ deserves to be further explored and improved. Although its carrier transport performance has been reported, few studies have investigated the effects of strain on this material. Strain effects on materials can tune its electronic band degeneracy and broaden its carrier transport properties. In the present study, we use DFT accompanied by Boltzmann transport theory and deformation potential theory to study the strain effect on the carrier transport properties of monolayer HfS_2_. The results indicate that the carrier effective mass is highly shrunken under the biaxial compressive strain reaches 4%. As a consequence, the carrier mobility of monolayer HfS_2_ reaches the highest value for both electron and hole. In this case, we surprisingly discovered that the highest mobility value was due to the carrier effective mass shrinking.

The effect of strain on the band structure and bandgap of monolayer HfS_2_ has been investigated in previous studies [11,12], showing that the application of tensile and compressive biaxial strain results in the bandgap increasing and decreasing, respectively. Manouchehr Hosseini et al. studied the effect of strain on the mobility of monolayer MoS_2_ and found that tensile biaxial strain could significantly enhance its mobility [13]; however, an investigation of strain effect on the mobility of other 1T-TMDs such as HfS_2_ was still missing. The conduction and valence band structures of HfS_2_ are different owing to indirect bandgap and the conduction band and valence band having different effective masses.

In this study, we present a comprehensive analysis of the effect of biaxial strain on the mobility of monolayer HfS_2_, employing the first principle method to perform band structure calculations and then the Kubo–Greenwood mobility approach for evaluating carrier mobility [14,15]. The methodology for the first principle method, compact band model, and Kubo–Greenwood mobility approach is briefly presented in Section 2. The effects of biaxial strain on the band structure and mobility of monolayer HfS_2_ are discussed in Section 3, and the concluding works are presented in Section 4.

## 2. Computational Methodology

This section explains the computational methodology for the evaluation of band structure, compact band model, and carrier mobility.

### 2.1. Band Structure of Monolayer HfS_2_

DFT calculations for determining the electronic structure and structure optimization of monolayer HfS_2_ were performed QuantumATK software (T-2022.03.) environment [16] by using a linear combination of the pseudoatomic orbital method and generalized gradient approximation (GGA) to describe the exchange–correlation functional. The plane wave energy cutoff was set to 500 eV and the Brillouin zone was sampled by an 8 × 8 × 1 mesh of Monkhorst–Pack *k* points. All the atomic positions were completely relaxed until the Hellmann–Feynman forces converged to 0.005 eV/Å and energy to 1meV. We adopted a 13 Å vacuum layer along the *z*-axis to avoid interaction between periodic images. Moreover, we also took into account the Heyd–Scuseria–Ernzerhof (HSE06) functional for the band structure calculation. The phonon spectrum was also obtained by QuantumATK. 

**Figure 1:**
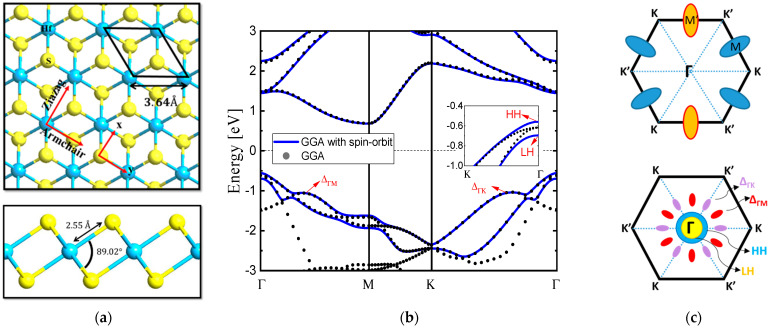
(**a**) Top and side (bottom) views of monolayer HfS_2_ showing a primitive hexagonal unit cell (Blue: Hf atoms; Yellow: S atoms) (**b**) Band structures of monolayer HfS_2_ along the high symmetric points in the hexagonal BZ. SOC splits the valence band HH and LH as shown in the inset. (**c**) Three lowest conduction band valleys are located at M’(M) points, six Δ_ГM_ (red) and six Δ_ГK_ (purple) bands with high symmetric points are located in the corresponding 1st BZ.

**Figure 2:**
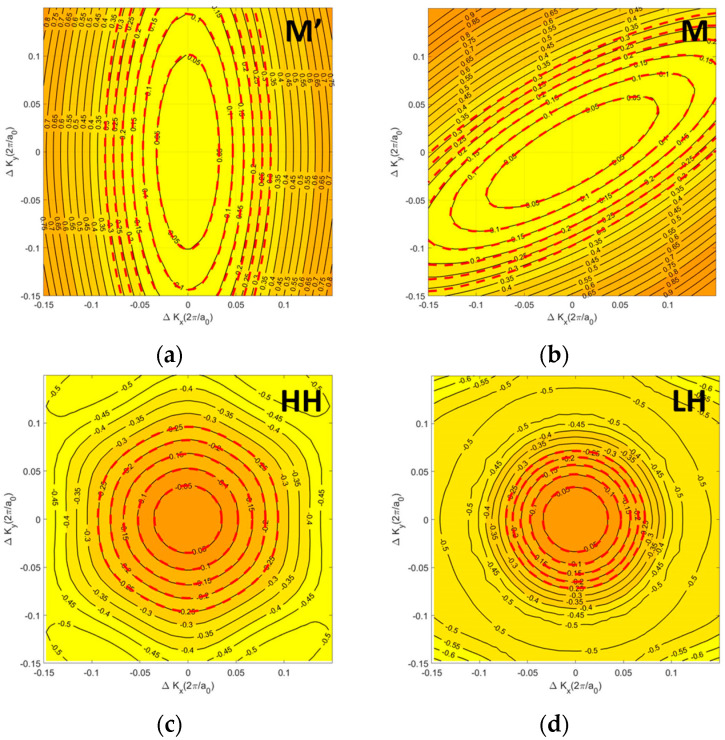
Energy contour plots of the (**a**) M’ valley, (**b**) M valley, (**c**) HH band, and (**d**) LH band of monolayer HfS_2_. Note that M’ and M valleys are in the CB while the HH and LH bands are in the VB. There energy contour plots are based on ab-initio band structure calculation performed using GGA with SOC. The dash lines are calculated results obtained using compact band models including the Rudenko model and new compact model.

Figure 1a shows the top and side views of atomic structure of the monolayer HfS_2_. The band structure of monolayer HfS_2_ and the corresponding first Brillouin Zone (1st BZ) are shown in Figure 1b. Monolayer HfS_2_ is a layered material composed of vertically stacked S–Hf–S layers through van der Waals forces. Each single S–Hf–S layer consists of two hexagonal planes of S atoms and an intermediated hexagonal plane of Hf atoms. Note that HfS_2_ is AB-stacked in HfS_2_. We constructed monolayer HfS_2_ structure by using the lattice parameter (*a* = 3.64 Å), which is close to the data presented in Reference [12]. The highly anisotropic CB of monolayer HfS_2_ with its minimum located at the M point is found in Figure 1b. The calculated indirect bandgap was 1.29 eV, in close agreement with the previous theoretical prediction [11]. There were three conduction band valleys in the 1st BZ of monolayer HfS_2_, as seen in Figure 1b. We extracted the electron effective masses of three M valleys in the two directions of armchair (AR) and zigzag (ZA) through parabolic fitting of the band structure, and these values are listed in Table 1. In the AR direction (i.e., M’–Г), valley M’ has a very heavy effective mass of m_c_ = 2.45m_0_ with a light transverse effective mass, while the other two M valleys have light ones of m_c_ = 0.31m_0_. In the other direction, ZA direction (i.e., M’–K), valley M’ has a light effective mass of m_c_ = 0.24m_0_ with a heavy transverse effective mass, and the other two M valleys show heavier effective masses of m_c_ = 0.74m_0_. The valence band structure of monolayer 1T-HfS_2_ along the Г–M and Г–K directions is also shown in Figure 1b, and the corresponding contour plots near the conduction band minima and valence band maximum are shown schematically in Figure 2. The CB minima at each M point are highly anisotropic, but the existence of three such valleys within the 1st BZ with rotational symmetry relaxes the overall direction dependence of transport, leading to similar monolayer HfS_2_ n-type-FET performance in the AR and ZA directions [7]. Conversely, the degenerate and nearly isotropic heavy hole (HH) and light hole (LH) VB peaks are located at the Г point (Figure 1b) according to ab-initio calculation with GGA only, and spin–orbit coupling (SOC) breaks the degenerate at the Г point and induced valence band HH and LH splitting via ab-initio calculation—performed using GGA and SOC. 

Figure 2 shows CB contour plots of unstrained HfS_2_ with the corresponding band structures around the M point as shown in Figure 1b. We can characterize two types of effective mass, namely the effective mass m_c_ in the transport direction and the density of states (DOS) effective mass, m_DOS_. We calculate m_c_ by fitting the curvature of the CB minimum/VB maxima in the transport direction. For example, the m_c_ of the M’ valley along the M–K direction (AR direction) equals m_MK_ and m_AR_. Details about m_c_ and m_DOS_ are presented in Table 1. The formula of m_DOS_ is followed by the definition from Reference [17]. In unstrained HfS_2_, the m_DOS_ of the valence band HH and LH are 0.47 m_0_ and 0.18 m_0_, respectively, as listed in Table 1. These values for the effective mass of the CB and VB of monolayer HfS_2_ are in agreement with the results of References [17,18]. In our paper, biaxial strain is defined as ε = (a − a_0_)/a_0_, where a_0_ and a denotethe equilibrium and deformed lattice constants, respectively. The effective mass of the carriers is calculated using the definition given in [19].

### 2.2. Compact Band Model

The band structure of both the CB and VB of monolayer HfS_2_ cannot be accurately described by using the simple effective mass approximation (EMA) model. First principle band structure was calculated using GGA deviates from the parabolic dispersion at low energies, which yielded the energy-dependent DOS. Here, to describe the E–K dispersion of the CB, we used the following implicit expression with energy-dependent effective mass (i.e., Rudenko model [20]),

(1) $$E=\frac{\hbar^{2}k_{x}^{2}}{2m_{x}\left(E\right)}+\frac{\hbar^{2}k_{y}^{2}}{2m_{y}\left(E\right)}$$

where *m_x_*(*E*) and *m_y_*(*E*) are energy-dependent functions. After converting the coordinate into a polar coordinate, Equation (1) can be rewritten as Equation (2):

(2) $$E=\frac{\hbar^{2}K^{2}cos^{2}\theta }{2m_{x}\left(E\right)}+\frac{\hbar^{2}K^{2}sin^{2}\theta }{2m_{y}\left(E\right)}$$

We have $E=\frac{\hbar^{2}k_{x}^{2}}{2\;m_{x}\left(E\right)}$ when θ=0°, we used $E\left(1+\alpha_{x}E\right)=\frac{\hbar^{2}k^{2}}{2m_{x0}}$ to fit the E–K relationship along the *x* direction and obtain the energy-dependent effective mass, *m_x_*(*E*),

(3) $$m_{x}\left(E\right)=m_{x0}\left(1+\alpha_{x}E\right)$$

For θ=90°,we define the energy-dependent effective mass, *m_y_*(*E*), as follows:

(4) $$m_{y}\left(E\right)=m_{y0}\left(1+\alpha_{y}E\right)$$

Finally, we transform Equation (2) to analytical form as follows:

(5) $$E=\left\{\frac{\hbar^{2}cos^{2}\theta }{2m_{x0}\left(1+\alpha_{x}E\right)}+\frac{\hbar^{2}sin^{2}\theta }{2m_{y0}\left(1+\alpha_{y}E\right)}\right\}K^{2}$$

Rearranging Equation (5) into the *K*(*E*, *θ*) form yields:

(6) $$K^{2}=\frac{E}{\left\{\frac{\hbar^{2}cos^{2}\theta }{2m_{x0}\left(1+\alpha_{x}E\right)}+\frac{\hbar^{2}sin^{2}\theta }{2m_{y0}\left(1+\alpha_{y}E\right)}\right\}}$$

We summarize the parameters used in the Rudenko model for the CB of monolayer HfS_2_ in Table 2.

For the VB, we adopted the new compact model to fit the first principle valence band structure of monolayer HfS_2_. To this end, the K(E) form of the new compact model, which is the opposite of the common E–K relationship, is used to possibly facilitate the successive calculation of other key physical quantities such as the DOS and energy contours for the VB. The form of the new compact model for HfS_2_ is expressed as follows:

(7) $$K\left(E,\theta \right)=A\left(E\right)+C\left(E\right)\times \cos\left(6\theta \right)$$

Two major parameters appear in the new compact model: First, the directionality (*θ*) help determine the direction of *k* moving towards various symmetry points. Second, the energy effect determined by the *A*(*E*) and *C*(*E*) functions. The form of functions *A*(*E*) and *C*(*E*) is the combination of k in different directions. Monolayer HfS_2_ presents six-fold symmetry, and the boundary condition is EMA-modelled with non-parabolic correction. Therefore, we have the following:

(8) $$E\left(1+\alpha_{1v}E\right)=\frac{\hbar^{2}k_{1}^{2}}{2m_{1v}}\;when\;K=k_{1}\;at\;\theta =0$$

(9) $$E\left(1+\alpha_{2v}E\right)=\frac{\hbar^{2}k_{2}^{2}}{2m_{2v}}\;when\;K=k_{2}\;at\;\theta =\frac{\pi }{6}$$

The form of *k*_1_ and *k*_2_ was acquiredfrom the boundary condition. In the equation, the coefficients of *α*, *β*, and *m_i_* (*i* = 1, 2) can be acquired by fitting with the first principle band structure. On the other hand, when *θ* = 0 or *π*/6, other relationship can be acquired as shown in Equations (10) and (11), respectively.

(10) $$k_{1}=A\left(E\right)+C\left(E\right)$$

(11) $$k_{2}=A\left(E\right)-C\left(E\right)$$

It can be further solves to obtain the relationship between key energy-dependent parameters such as *A*(*E*) and *C*(*E*) with *k*_1_ and *k*_2_.

(12) $$A\left(E\right)=\frac{1}{2}\left[k_{1}+k_{2}\right]$$

(13) $$C\left(E\right)=\frac{1}{2}\left[k_{1}-k_{2}\right]$$

We summarize the parameters used in the new compact model for the VB of monolayer HfS_2_ in Table 3.

Finally, the Rudenko model and the new compact model are in the form of *K*(*E*, *θ*), and they can be used to calculate the DOS per spin:

(14) $$D\left(E\right)=\frac{1}{{\left(2\pi \right)}^{2}}\int_{0}^{2\pi }K\left(E,\theta \right)\frac{d}{dE}K\left(E,\theta \right)\;d\theta$$

Reference [11] was followed to define the *x* component of the group velocity square *V_x_*^2^(*E*):

(15) $$V_{x}^{2}\left(E\right)=\;\frac{\frac{1}{4\pi^{2}}\int_{0}^{2\pi }V_{x}^{2}K\left(E,\theta \right)\frac{\partial K\left(E,\theta \right)}{\partial E}d\theta }{\frac{1}{4\pi^{2}}\int_{0}^{2\pi }K\left(E,\theta \right)\frac{\partial K\left(E,\theta \right)}{\partial E}d\theta }$$

### 2.3. Kubo–Greenwood Formula for Carrier Mobility

According to the compact band structure as introduced in the previous subsection, we calculated the carrier transport using the Kubo–Greenwood mobility formula [14,15]. The parameters used to calculate the carrier mobility of monolayer HfS_2_ are presented in Table 4. This calculation uses the momentum relaxation time approximation according to the Kubo–Greenwood formula [14,15]. The scattering mechanisms included in this calculation contain the phonon scatterings, such as acoustic phonon and optical phonon. Once the relaxation times of the phonon-limited scattering mechanisms were determined, the carrier mobility formula for each band, shown as follows, was derived from the Kubo–Greenwood formula.

(16) $$\mu_{xx}=\frac{e\int_{0}^{\infty }D\left(E\right)\;V_{x}^{2}\left(E\right)\;\tau \left(E\right)\;f\left(E\right)\;\left[1-f\left(E\right)\right]\;dE}{k_{B\;}T\;\int_{0}^{\infty }D\left(E\right)\;f\left(E\right)\;dE}=\frac{e\langle \tau \rangle }{m_{c}}$$

where *f*(*E*) denotes the Fermi–Dirac function, *E* is the total energy, *V_x_*^2^(*E*) is the energy-dependent group velocity square along the *x* direction, *τ*(*E*) is the scattering time, and *D*(*E*) is the density of states. *E*_0_ is the band edge energy. The scattering time is determined by summing all scattering rates, considered:

(17) $$\frac{1}{\tau \left(E\right)}=\frac{1}{\tau_{ADP}\left(E\right)}+\frac{1}{\tau_{ODP}\left(E\right)}+\frac{1}{\tau_{POP}\left(E\right)},\;\mathrm{where}$$

(18) $$\frac{1}{\tau_{ADP}\left(E\right)}=\frac{2\pi }{\hbar }\left(\frac{D_{ac}^{2}k_{B}T}{\rho v_{s}^{2}}\right)D\left(E\right)$$

(19) $$\frac{1}{\tau_{ODP}\left(E\right)}=\frac{2\pi }{\hbar }\left(\frac{\hbar D_{op}^{2}}{2\rho \omega_{op}}\right)\left(N_{q}D\left(E+\hbar \omega_{op}\right)+\left(N_{q}+1\right)D\left(E-\hbar \omega_{op}\right)\right)$$

where *τ_ADP_*(*E*) and *τ_ODP_*(*E*) denote the scattering times of acoustic phonon and optical phonon, respectively. N_q_ is the Bose occupation of phonon *ω_op_*. *D_ac_* and *D_op_* denote the deformation potential of acoustic and optical phonon, respectively. In monolayer HfS_2_, polarization due to the lattice vibration of the polar LO phonon is oriented along the plane of the layer. The microscopic approach based on the atomic Born effective charges is used, and scattering rate formula, *τ_POP_*(*E*), for polar LO–phonon interaction in monolayer HfS_2_ is given in the literature [6,21,22].

Assuming the scattering time *τ*(*E*) to be independent of the energy, *E*, it is derived from Equation (16) to obtain the conductivity effective mass *m_c_* [23,24]:

(20) $$m_{c}=\frac{k_{B}T\int_{0}^{\infty }D\left(E\right)\;f\left(E\right)\;dE}{\int_{0}^{\infty }D\left(E\right)\;V_{x}^{2}\left(E\right)f\left(E\right)\left[1-f\left(E\right)\right]\;dE}$$

It is straightforward to use the right hand side of Equations (16) and (20) to obtain the average scattering time, <*τ*>.

## 3. Results and Discussion

In unstrained monolayer HfS_2_, the lowest minima in the CB are denoted as M’ valley and M valley in Figure 1b. One M’ valley and two M valleys are degenerate in the unstrained and biaxial strain conditions, respectively. However, upon the application of biaxial strain, they do not split into one M’ valley and two M valleys with different effective masses and energy minima. For the VB of the unstrained monolayer HfS_2_, the top two bands are denoted as the HH band and LH band, respectively. Ab-initio band structure calculation using GGA with SOC. We further investigated the band structure close to the CB and VB via contour plot, which shows the constant energy lines as shown in Figure 2. The CB resembles a valley with a parabolic elliptic energy dispersion, known as the M’ and M valleys. For the valence bands, the LH looks like an isotropic parabolic band while the HH is a six-fold symmetry band. The contours around the HH and LH bands are almost isotropic and circular at low energy, whereas the M’ and M valleys demonstrate anisotropic behavior. While the valence bands, including the HH and LH, show almost isotropic dispersion, a strong anisotropic one occurs in the CB around the M’ and M points owing to the difference in the orbital structures of the VB (p orbit of S atom) and CB (d orbit of Hf atom).

**Figure 3:**
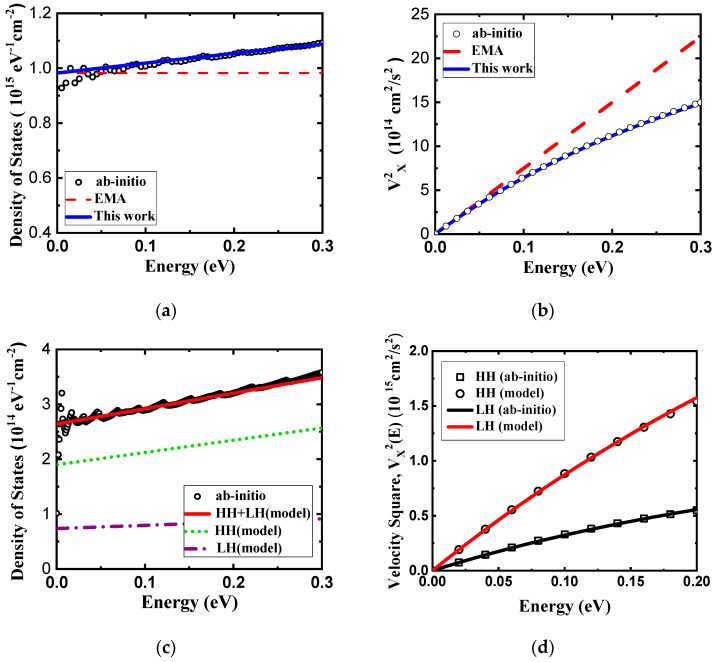
(**a**) Density of states and (**b**) velocity square as function of energy for the M’ valley of monolayer HfS_2_. (**c**) Density of states and (**d**) velocity square as function energy for the valence band of monolayer HfS_2._ These calculations are based on ab-initio GGA band structure.

In Figure 3, we present the DOS and velocity square of monolayer HfS_2_, as obtained using the compact band models and ab-initio calculations. Our model fits very well with the ab-initio results, owing to the use of an energy-dependent effective mass function. Our model is better than the EMA model (which used a constant effective mass) for carrier mobility calculation.

**Figure 4:**
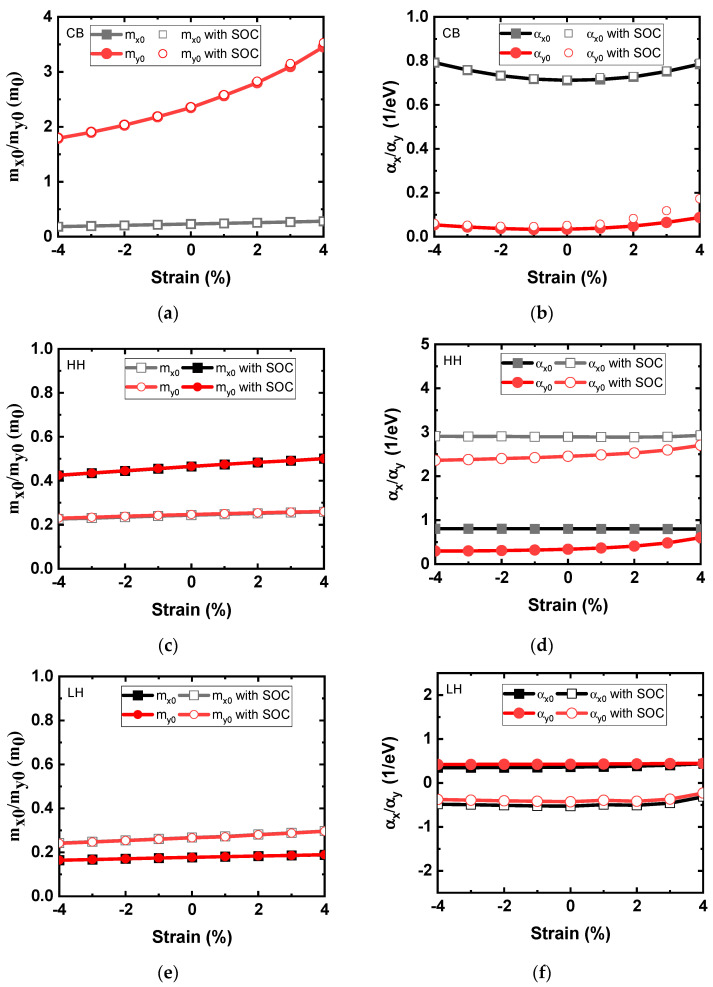
(**a**) Effective mass and (**b**) non-parabolicity factor of the Rudenko model for the CB of monolayer HfS_2_ under biaxial strain. (**c**) Effective mass and (**d**) non-parabolicity factor of the new compact model for the valence band HH of monolayer HfS_2_ under biaxial strain. (**e**) Effective mass and (**f**) non-parabolicity factor of the new compact model for the valence band LH of monolayer HfS_2_ under biaxial strain.

The key parameters including the effective masses and non-parabolicity factors of the CB and VB compact models under biaxial strain are shown in Figure 4. The effective mass and the non-parabolicity factor of the Rudenko model are extracted from the DFT-calculated band structure and reported in Figure 4a and Figure 4b, respectively. In the CB, two paths in the M’ valley have different effective masses. We denote the effective mass in the armchair direction (M’–Г) as *m_y_*_0_ and the effective mass along the zigzag direction (i.e., M’–K) as *m_x_*_0_. As illustrated in the figure, the *m_y_*_0_ in the M’ valley is ~10× larger than *m_x_*_0_. The non-parabolicity factor α_y_ is extremely small compared to α_x_, and it slightly increases as tensile strain increases. The difference of two effective masses in the M’ valley increases as the strain increases, and the M’ valley exhibits more anisotropy under tensile strains. Meanwhile, the effective mass of the M’ valley decreases as the strain increases and becomes the lowest effective mass at a compressive biaxial strain of 4%. In Figure 4c,d, the key parameters of the new compact band model are also extracted from the ab-initio valence band HH structure. In Figure 4e,f, the key parameters of the new compact band model are also extracted from the ab-initio valence band LH structure. The effective mass decreases as biaxial compressive strain increases for the CB and VB models, while it increases as biaxial tensile strain increases. The hole effective mass was explored under various strains. Two HH and LH bands contribute to the major VB over most of the applied strain range. The HH and LH exhibit the lowest effective mass under compressive biaxial strain, and the top VB corresponds to the HH band in this strain range. The effective mass of the HH band increases as the biaxial strain changes from a compressive to the tensile regime.

**Figure 5:**
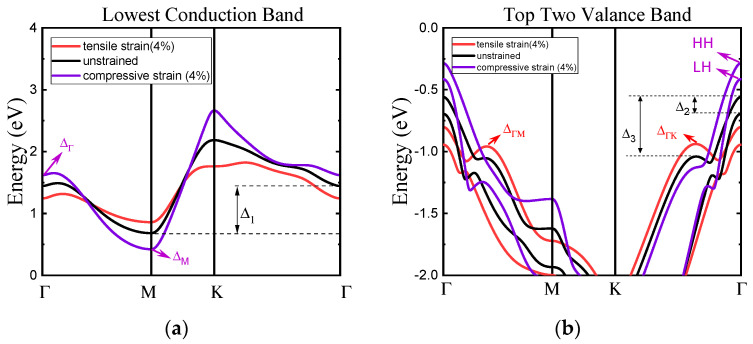
Changes in (**a**) the lowest CB and (**b**) the two highest VBs of monolayer HfS_2_ under compressive and tensile biaxial strains as calculated using ab-initio method with GGA considering SOC.

Figure 5 illustrates the band structure of the unstrained and strained monolayer HfS_2_, including the lowest M valley in the CB and the two highest VBs (HH and LH). The band structure is plotted for five strains: −4%, −2%, 0%, 2%, and 4%. The M valley HfS_2_ is lower than the other valleys and located at the conduction band minimum (CBM). Note that the M valley is still dominant across all strain regions. As can be inferred from this figure, the strain can modify the shape and energy of each valley. Under biaxial strain, the CB and the VB of the edges shift upward and downward as strain increases, respectively. The energy distance between the M valley and the Г valley of unstrained material is evaluated to be ~720 meV, which is consistent with the values reported in References [4,11]. Biaxial compressive strain increases this energy distance, tensile strain decreases it. This implies that the effective mass along the M’–Г direction decreases with increasing biaxial compressive strain. For the VBs, the HH and LH energy splitting at the Г point is due to SOC not being sensitive to strain. In particular, a relatively large biaxial compressive strain shifts the band edge energy of the HH band so that it becomes the top VB, as shown in Figure 5b. Here we can anticipate that, while under compressive strain, one can neglect the scattering between the HH band and the Δ_ГM_ and Δ_ГK_ bands, and, under tensile strain, this type of scattering can significantly degrade hole mobility.

**Figure 6:**
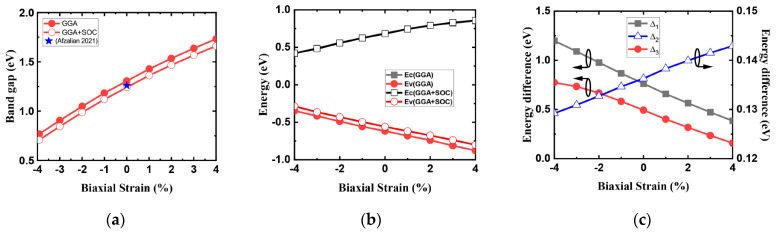
(**a**) The variation in the bandgap of monolayer HfS_2_ under biaxial strain [6]. (**b**) The absolute conduction band edge (*Ec*) and valence band edge (*Ev*) for ab-initio calculations with GGA with and without SOC a function of biaxial strain. (**c**) Energy difference between the conduction band (Δ_1_) and the valence band valleys (Δ_2_ and Δ_3_) versus biaxial strain.

Strain ranging from −4% to +4% is applied to the band structure of monolayer HfS_2_ in Figure 6. The bandgap of monolayer HfS_2_ as a function of biaxial strain is shown in Figure 6a. The bandgap of unstrained HfS_2_ is 1.25 eV, and in agreement with References [3,6,25,26]. The strain dependence of the bandgap from our work is in agreement with References [1,11,12]. It is well known that strain can modify band structure. In this study, we found that biaxial tensile strain led to an increase in the bandgap, and that biaxial compressive strain led to a decrease in the bandgap. Under the biaxial stress effect es of −4 to 4%, the bandgap of the system increases from 0.65 to 1.67 eV. In Figure 6b, the absolute conduction band edge (*E_c_*) and the valence band edge (*E_v_*) for ab-initio calculations performed using GGA with and without SOC as a function of the biaxial strain. *E_c_* denotes the M valley and *E_v_* denotes the HH band. The energy at the bottom (top) of CB (VB) increases (decreases) with respect to strain increase. Both *E_c_* and *E_v_* almost change linearly with respect to biaxial strain, but the slope is opposite. SOC will caused an upward shift in the energy of *E_v_*. Moreover, the strain-induced modulation of *E_c_* was relatively smaller than that of *E_v_*. Figure 6c shows the variation in energy difference between the Г and M valleys denoted as Δ_1_, and the valence band difference between the HH band and the Δ_ГK_ band (Δ_3_) and the HH–LH band splitting (Δ_2_) under different biaxial strains, respectively.

**Figure 7:**
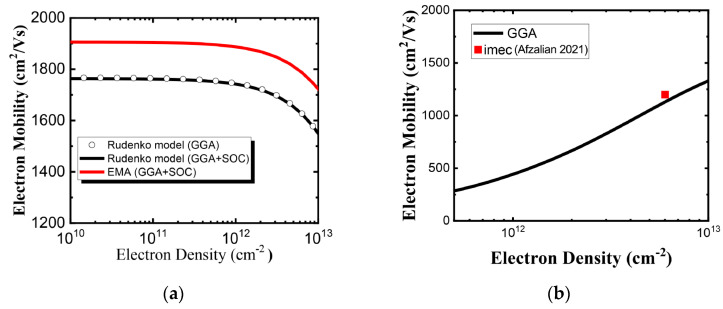
(**a**) Phonon-limited electron mobility without POP scattering versus electron density with two compact band models for monolayer HfS_2_. (**b**) Electron mobility with POP scattering as a function of electron density [6].

**Figure 8:**
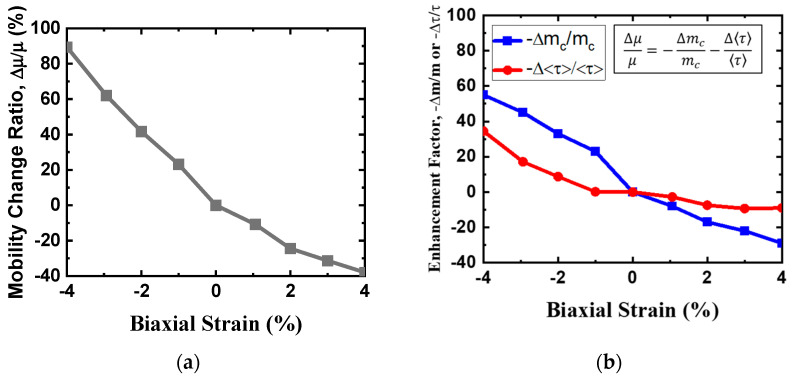
(**a**) Electron mobility change ratio as a function of biaxial strain. (**b**) Enhancement factors ($-\frac{\Delta m_{c}}{m_{c}}$ and $-\frac{\Delta \tau }{\tau }$) versus biaxial strain.

According to Reference [6], dynamic doping with a gate voltage of the 2D FET electrostatically induces a high carrier concentration and maintains the high carrier mobility of monolayer HfS_2_. We used a carrier density of 6 × 10^12^ cm^−2^ in the following results for calculated carrier mobility. Figure 7a shows the room temperature electron mobility without polar optical phonon (POP) scattering versus the electron density for the unstrained monolayer HfS_2_ in two compact models. The calculated electron mobility calculated using the Rudenko model is lower than that using EMA model owing to the conduction band non-parabolicity effect. This result implies that the non-parabolicity effect is important and cannot be neglected in electron mobility calculation. The SOC causes almost no change in the CB according to the ab-initio GGA calculation, while the result in calculated electron mobilities identical to those obtained using the Rudenko model which fits the GGA ab-initio conduction bands with and without SOC. At low carrier concentrations, mobility is strongly limited by POP scattering. However, as the carrier concentration increases, the screening effect reduces the impact of POP scattering. As can be seen in Figure 7b, the electron mobility with POP scattering increases with the electron density for monolayer HfS_2_ because of screening, and our calculations are in agreement with imec’s work [6]. Square symbol is imec’s calculated data from Reference [6]. The biaxial strain dependency of the intrinsic phonon-limited mobility is presented in Figure 8a. Enhancement factors due to −Δ*m*/*m* and −Δ*τ*/*τ* versus biaxial strain are shown in Figure 8b.

Apparently, the effects of compressive and tensile strain on electron mobility are distinct, and this difference can be mainly be attributed to the roles of electron effective mass and scattering rate. For example, under biaxial compressive strain, the electron effective masses of the M’ and M valleys are much lower compared to the unstrained condition. A reduced equivalent effective mass *m_c_* implies a decrease in the density of states. According to Equation (19), the scattering time increases owing to the reduced density of states, which in turn enhances electron mobility. Under biaxial tensile strain, however, the electron effective mass of the CB higher is higher than that of the unstrained one. With a biaxial compressive strain of 4%, the phonon-limited mobility becomes 90% higher than that of the unstrained material. In contrast, a biaxial tensile strain of 4% reduces the electron mobility by ~40% as compared to that under the unstrained condition, owing to the increase in both effective mass and scattering rate. Moreover, Figure 8 indicates that the biaxial strain-induced electron mobility enhancement with compressive strain is larger than that with tensile strain.

**Figure 9:**
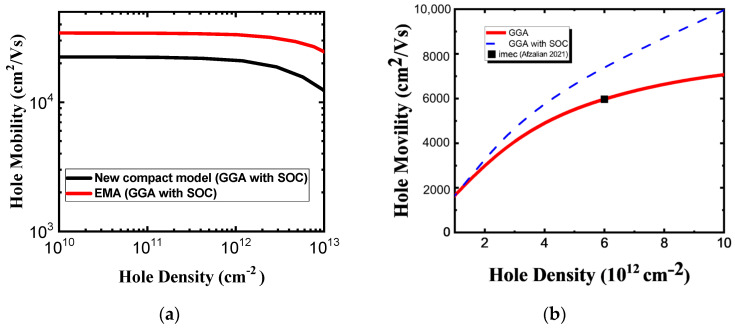
(**a**) Phonon-limited hole mobility without POP scattering versus hole density with two compact band models for monolayer HfS_2_. (**b**) Hole mobility with POP scattering as a function of hole density.

**Figure 10:**
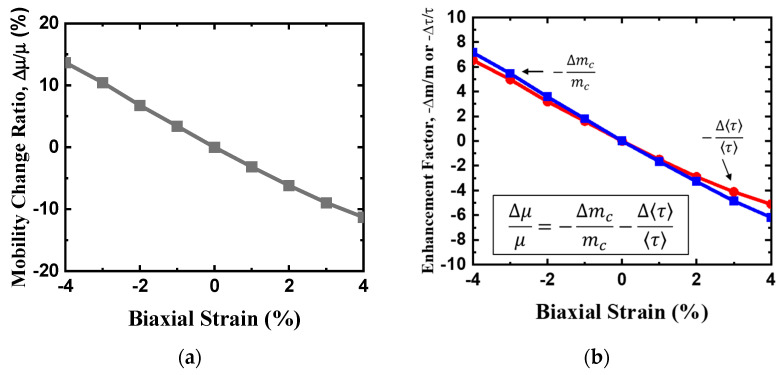
(**a**) Hole mobility change ratio as a function of biaxial strain. (**b**) Enhancement factors ($-\frac{\Delta m_{c}}{m_{c}}$ and $-\frac{\Delta \tau }{\tau }$) versus biaxial strain.

Figure 9a shows the room temperature hole mobility without POP scattering versus hole density for the unstrained monolayer HfS_2_ in two compact models. As can be inferred from these plots,, the hole mobility determined using the new compact band model is lower than that determined using the EMA model, owing to the valence band non-parabolicity effect. The non-parabolicity effect in the band structure alters the carrier dynamics within the band, which in turn affects carrier mobility and electrical conductivity. This implies that the non-parabolicity effect is important in hole mobility calculation and cannot be ignored. The enhanced screening effect of POP scattering weakens the electrostatic interactions between holes. As illustrated in Figure 9b, the hole mobility with POP scattering increases with the hole density for monolayer HfS_2_ because of screening, and our calculations are in agreement with the imec’s work [6]. Square symbol is imec’s calculated data from Reference [6]. Note that SOC strongly affects HH and LH valence band degeneracy at the Г point and causes valence band splitting. The calculated hole mobility calculated using the new compact model, which fits the GGA ab-initio valence band structure with SOC, is higher than the one without SOC owing to the HH and LH band splitting effect. Most holes are in the HH band in the GGA ab-initio valence band structure, and with effective mass shrinkage of the HH due to SOC. The findings from the calculations for hole mobility can be attributed to these trends, as shown in Figure 9b. The biaxial strain-dependency of the intrinsic phonon-limited hole mobility change ratio is presented in Figure 10a. The enhancement factors of hole mobility due to −Δ*m_c_*/*m_c_* and −Δ<*τ*>/<*τ*> versus biaxial strain are shown in Figure 10b. The relationship between the hole mobility change ratio and the enhancement factors is included. Apparently, the effects of compressive and tensile biaxial strain on hole mobility are remarkably different, which can mainly be attributed to the role of hole effective mass and scattering rate. For example, under biaxial compressive strain, the effective masses of the HH and LH bands are much lower than in an unstrained condition, which enhances hole mobility. By contrast, under biaxial tensile strain, the hole effective mass of VB is higher than that of the unstrained one. In the biaxial strain range of −4% to 4%, hole inter-band scattering due to the HH band and satellite bands such as Δ_ГM_ and Δ_ГM_ is not serious and can be ignored. This is because high mobility generally means that carriers can traverse longer distances within the material (with a longer mean free path) without being affected by scattering. Consequently, the probability of carriers interacting with defects, lattice vibrations, or other scattering in the material is decreased. Moreover, when biaxial tensile strain is >4%, it increases the valence band degeneracy of monolayer HfS_2_ even more, and the degenerate valence bands and higher inter-band phonon scattering rate reduce the hole mobility. Under biaxial compressive strain, the hole mobility increases because of the reduction in the hole effective mass and results in the reduction of the scattering rate. Under a biaxial compressive strain of 4%, the phonon-limited mobility increases by ~13% compared to that of the unstrained material. In contrast, a biaxial tensile strain of 4%, the hole mobility decreases by ~11% compared to that of the unstrained, owing to increases in the hole effective mass and scattering rate.

## 4. Conclusions

We examined the carrier transport performances of monolayer HfS_2_ through ab-initio calculation, compact band model, and the Kubo–Greenwood mobility approach. This study revealed that SOC has a substantial and non-negligible impact on the carrier transport properties of HfS_2_. We proposed two simple yet accurate compact models to analyze the CB and VB of HfS_2_, using energy-dependent effective masses to characterize the material properties. The dependency of carrier mobility performances on strain engineering is assessed and benchmarked against the carrier mobility of the unstrained materials. The results indicated that, under a compressive biaxial strain reaches 4%, the carrier mobility exhibits the maximum value. As a result, the highest electron and hole mobility values under the 4% compressive biaxial strained case are ~1.9 and ~1.1 times greater, respectively, than those of the unstrained one. These findings offer physical insights into the tuning of carrier transport properties for monolayer HfS_2_.

## Figures and Tables

**Table 1:** The effective mass for CB and VB of monolayer HfS_2_ as determined by ab-initio calculation with GGA and GGA + SOC, respectively.

**GGA**			
Valley/band	m_MГ_/m_AR_ (m_0_)	m_MK_/m_ZA_ (m_0_)	m_DOS_ = (m_MГ_m_MK_)^1/2^ (m_0_)
M’ valley	2.36	0.23	0.74
M valley	0.3	0.71	0.74
	m_ГM_ (m_0_)	m_GK_ (m_0_)	m_DOS_ = (m_ГM_m_ГK_)^1/2^ (m_0_)
HH band	0.47	0.47	0.47
LH band	0.18	0.18	0.18
**GGA + SOC**			
Valley/band	m_MГ_/m_AR_ (m_0_)	m_MK_/m_ZA_ (m_0_)	m_DOS_ = (m_MГ_m_MK_)^1/2^ (m_0_)
M’ valley	2.35	0.23	0.74
M valley	0.3	0.71	0.74
	m_ГM_ (m_0_)	m_GK_ (m_0_)	m_DOS_ = (m_ГM_m_ГK_)^1/2^ (m_0_)
HH band	0.25	0.24	0.24
LH band	0.27	0.27	0.27

**Table 2:** The new compact model parameters for the CB of monolayer HfS_2_.

**GGA**		
θ	$m\;\left(m_{0}\right)$	$\alpha \;\left(1/\mathrm{eV}\right)$
0	$m_{1c}=0.23$	$\alpha_{1c}=0.71$
π/2	$m_{2c}=2.36$	$\alpha_{2c}=0.03$
**GGA + SOC**		
θ	$m\;\left(m_{0}\right)$	$\alpha \;\left(1/\mathrm{eV}\right)$
0	$m_{1c}=0.23$	$\alpha_{1c}=0.71$
π/2	$m_{2c}=2.35$	$\alpha_{2c}=0.05$

**Table 3:** The new compact model parameters for the HH/LH VB of monolayer HfS_2_.

**GGA**		
θ	$m\;\left(m_{0}\right)$ HH/LH	$\alpha \;\left(1/\mathrm{eV}\right)$ HH/LH
0	$m_{1v}=0.47/0.18$	$\alpha_{1v}=0.36/0.43$
π/6	$m_{2v}=0.47/0.18$	$\alpha_{2v}=0.85/0.36$
**GGA + SOC**		
θ	$m\;\left(m_{0}\right)$ HH/LH	$\alpha \;\left(1/\mathrm{eV}\right)$ HH/LH
0	$m_{1v}=0.25/0.27$	$\alpha_{1v}=2.5/-0.43$
π/6	$m_{2v}=0.24/0.27$	$\alpha_{2v}=2.9/-0.53$

**Table 4:** The effective mass, deformation potential constant of electron and hole for unstrained monolayer HfS_2_ by ab initio calculation with GGA.

Carriers	m_MГ/_m_HH_ (m_0_)	m_MK_/m_LH_ (m_0_)	*D_ac_* (eV)	*D_op_* (10^8^ eV/cm)
electron	2.36	0.23	1.31	0.99
hole	0.47	0.18	0.95	0.73

## Data Availability

Data are contained within the article.
